# Changes in GPS-Derived Community Mobility After Motor Skill Training in Older Adults

**DOI:** 10.1093/geroni/igab046.2163

**Published:** 2021-12-17

**Authors:** Pamela Dunlap, Breanna Crane, Kyle Moored, Michelle Carlson, Subashan Perera, Jennifer Brach, Brooke Klatt, Andrea Rosso

**Affiliations:** 1 University of Pittsburgh, Pittsburgh, Pennsylvania, United States; 2 Johns Hopkins Bloomberg School of Public Health, Baltimore, Maryland, United States; 3 Johns Hopkins University, Baltimore, Maryland, United States; 4 University of pittsburgh, Pittsburgh, Pennsylvania, United States

## Abstract

The study purpose was to identify the effects of a motor skill training intervention to improve gait speed on community mobility among community-dwelling older adults. The study included 249 participants randomized to standard physical therapy or a standard plus motor skill training program. Community mobility was measured using the Life Space Assessment (LSA) and GPS at baseline, 12 (post-intervention), 24 and 36 weeks. There were 124 participants (M age=77.4±6.7; 68.6% female; LSA: 76.2±17.6) randomized to the standard plus and 125 (M age=77.4±6.4; 62.4% female; LSA: 74.3±18.2) to the standard group. There was no significant between-group difference in pre- or post-intervention LSA scores and no significant pre- to post-intervention change over time in either group. GPS results are pending. While there were no differences in self-reported LSA, we anticipate objective GPS measurement of community mobility will better capture post-intervention changes and differences between groups.

